# 2064: Deconstructing the peptide specificity of TCR recognition

**DOI:** 10.1017/cts.2017.195

**Published:** 2018-05-10

**Authors:** Timothy P. Riley, Juan Mendoza, K. C. Garcia, Brian M. Baker

**Affiliations:** University of Notre Dame, Notre Dame, IN, USA

## Abstract

OBJECTIVES/SPECIFIC AIMS: The off-target and organ-specific toxicities observed in cancer immunotherapy present an obstacle to T-cell-based therapeutics. A recent clinical trial underscored the need for improved methods to define TCR specificity after melanoma patients treated with TCR engineered T-cells suffered from fatal cardiovascular toxicity arising from the unpredicted recognition of a muscle-specific peptide. METHODS/STUDY POPULATION: To address this drawback to T-cell-based immunotherapies, we developed a novel protein engineering approach to define the peptide specificity of a given TCR. Here, directed evolution in a yeast display system produced a large scale peptide library, where recognition by the melanoma reactive DMF5 TCR acted as the guiding selective pressure. After this technique identified a panel of putative cross reactive peptides, sequence analysis and computational modeling followed by kinetic binding experiments and structural analysis determined the DMF5 TCR recognizes 2 distinct classes of peptides through chemically distinct mechanisms. RESULTS/ANTICIPATED RESULTS: This information led to the rational, structure-based design of additional cross reactive peptides and introduced a unique approach to screen the human proteome and identify the TCR targets which triggered undesired autoimmunity when this molecule was used in clinical trials. DISCUSSION/SIGNIFICANCE OF IMPACT: The distinct chemical nature of the 2 peptide classes suggest TCRs are more cross reactive than previously thought, presenting an obstacle to cell-based immunotherapy. Defining the peptide specificity of TCRs is of high interest to the immunology community, and will lead to improved approaches to designing engineered TCRs for cell therapy.

